# Effects of Ketamine Administration on Auditory Information Processing in the Neocortex of Nonhuman Primates

**DOI:** 10.3389/fpsyt.2020.00826

**Published:** 2020-08-19

**Authors:** Misako Komatsu, Noritaka Ichinohe

**Affiliations:** ^1^ Laboratory for Molecular Analysis of Higher Brain Functions, RIKEN Center for Brain Science, Saitama, Japan; ^2^ Department of Ultrastructural Research, National Center of Neurology and Psychiatry, Tokyo, Japan

**Keywords:** electrocorticography, common marmoset, monkey, tonotopy, offset response, ECoG

## Abstract

Ketamine, an N-methyl-D-aspartate (NMDA) receptor antagonist, exerts broad effects on consciousness and perception. Since NMDA receptor antagonists induce cognitive impairments, ketamine has been used for translational research on several psychiatric diseases, such as schizophrenia. Whereas the effects of ketamine on cognitive functions have been extensively studied, studies on the effects of ketamine on simple sensory information processing remain limited. In this study, we investigated the cortex-wide effects of ketamine administration on auditory information processing in nonhuman primates using whole-cortical electrocorticography (ECoG). We first recorded ECoG from awake monkeys on presenting auditory stimuli of different frequencies or different durations. We observed auditory evoked responses (AERs) across the cortex, including in frontal, parietal, and temporal areas, while feature-specific responses were obtained around the temporal sulcus. Next, we examined the effects of ketamine on cortical auditory information processing. We conducted ECoG recordings from monkeys that had been administered anesthetic doses of ketamine from 10 to 180 min following administration. We observed significant changes in stimulus feature-specific responses. Electrodes showing a frequency preference or offset responses were altered following ketamine administration, while those of the AERs were not strongly influenced. However, the frequency preference of a selected electrode was not significantly altered by ketamine administration over time following administration, while the imbalances in the onset and offset persisted over the course of 150 min following ketamine administration in all three monkeys. These results suggest that ketamine affects the ability to distinguish between sound frequency and duration in different ways. In conclusion, future research on the NMDA sensitivity of cortical wide sensory information processing may provide a new perspective into the development of nonhuman primate models of psychiatric disorders.

## Introduction

Ketamine, an N-methyl-D-aspartate (NMDA) receptor antagonist, exerts broad effects on consciousness and perception. Several reports have documented that ketamine induced dissociative and extracorporeal sensations when patients recovered from ketamine-induced anesthesia (1–3). Furthermore, a subanesthetic dose of ketamine induces illusions and alterations in hearing, vision, or proprioception in healthy human volunteers, as well as psychotomimetic effects, such as hallucinations, paranoia, formal thought disorder, and cognitive impairment [e.g., (4)]. Based on reports where NMDA receptor antagonists induce cognitive impairments in nonhuman primates (NHP) (5–10), a subanesthetic dose of ketamine has been used for translational research on schizophrenia (6, 9, 11), Alzheimer’s disease (12), and autism spectrum disorder (8). These studies suggested that ketamine induces prefrontal cortical dysfunction, while recent studies reported aberrant neuronal oscillations in the prefrontal cortex of NHP (13, 14).

In addition to cognitive impairments, several studies on humans, nonhuman primates, and rodents have examined the effects of a subanesthetic dose of ketamine on neurophysiological indices of sensory information processing. Especially, auditory steady-state response (ASSR) (15, 16) and mismatch negativity (MMN) [e.g. (17, 18)] have been studied as more robust probes of abnormal auditory information processing in schizophrenia. ASSR is an electrophysiological response entrained to the frequency and the phase of periodic auditory stimuli, and is most evident when stimuli are presented in the gamma frequency range 30–50 Hz in humans (19). Alteration of ASSR has reported various neuropsychiatric illness, and is considered to reflect cortical excitation/inhibition imbalance (16). These dysfunctional oscillations may arise owing to anomalies in the networks of gamma-aminobutyric acidergic interneurons and pyramidal neurons (20, 21). Furthermore, recent animal studies suggested that ASSR modulations by ketamine is dose dependent (22, 23). On the other hand, MMN is an event-related potential (ERP) elicited most commonly in the context of auditory oddball paradigms [e.g., (24)], and is considered to reflect neural activity for deviance detections. Gil-da-Costa, Stoner (25) reported reduced MMN in monkeys following the subanesthetic administration of ketamine, as found in humans with schizophrenia (26, 27), and in healthy adult/young subjects having been administered ketamine (4, 28). These results based on electroencephalograms (EEGs) suggest that ketamine induces changes in neural activity related to early auditory information processing in humans and NHPs, like in schizophrenia patients. Furthermore, electrophysiological studies with depth electrodes reported that the primary auditory area of NHPs showed mismatch-related activity, which was inhibited by NMDA receptor antagonists (29, 30). Whereas auditory indices, ASSR and MMN, have been extensively studied, the effects of ketamine on auditory information processing itself have only been studied to a limited degree (28, 31).

In this study, we hypothesized ketamine affects even simple auditory feature processing (e.g., frequency or duration), and investigated the cortex-wide effects of ketamine administration by exposing common marmosets to auditory stimuli of different frequencies or durations. The marmoset, a small new-world monkey, has an anatomical organization homologous to that of humans and macaques (32, 33) and has been reported to have MMN-like auditory responses (34). Their lissencephalic brains enable direct access to the core auditory areas. The ECoG array covers a whole hemisphere of the marmoset from the frontal pole to the occipital and temporal poles. The array enables us to observe neuronal activity from an entire hemisphere with high spatiotemporal resolution.

## Materials and Methods

This study was carried out in accordance with the recommendations of the National Institutes of Health Guidelines for the Care and Use of Laboratory Animals. The protocol was approved by the RIKEN Ethical Committee (No. H28-2-221 (3)). All surgical procedures were performed under anesthesia, and all efforts were made to minimize the number of animals used, as well as their discomfort. In so doing, we sought to deliver the most humane care and treatment possible. Parts of the dataset are shared on the public server Brain/MINDS Data Portal (brainminds.riken.jp).

### Subjects

We used five adult common marmosets (*Callithrix jacchus*; three males and two females, 320–470 g, 14–53 months). Before the ECoG arrays were implanted into the monkeys, they were familiarized with the experimenter and experimental settings. The animals had *ad libitum* access to food and water throughout the experimental period. Three of these animals were used for experiments with ketamine administration.

### Implantation of ECoG Array

The whole-cortical 96ch ECoG arrays (Cir-Tech Co. Ltd., Japan) (35) were chronically implanted and used for neural recordings. We epidurally implanted the array into the right (monkey O, R, and S) or left (monkey J and M) hemisphere of each monkey. Fifteen and three electrodes from monkeys M and R, respectively, were cut during the implantation. The surgical procedures for electrode implantation have been previously described in detail (35). Following the animals’ recovery, the positions of each electrode contact were identified based on computer tomography, and aligned to pre-acquired T2-weighted anatomical magnetic resonance images using AFNI software (36) (http://afni.nimh.nih.gov). Finally, we estimated the location of each electrode on cortical areas by registering a marmoset brain atlas (37) to the MRI with AFNI and ANTS (38). Based on putative cortical areas, we divided the electrodes into six groups, consisting of the prefrontal, sensorimotor, posterior parietal, visual, temporal, and auditory areas. In all monkeys, the electrode array covered the frontal, parietal, occipital, and temporal cortices (**Supplementary Figure S1** and **Supplementary Table S1**).

### Auditory Stimuli

We used auditory stimuli of different durations (AD) and frequencies (AF). In AD, 10 types of pure sinusoidal tones (1 ms rise/fall) with different durations (10, 25, 50, 75, 100, 125, 150, 175, 200, and 225 ms; 1,000 Hz; 2,000 stimuli in total) were randomly presented with an equal probability of 10%. In AF, 10 types of tones with different frequencies (700, 800, 900, 1,000, 1,100, 1,200, 1,300, 1,400, 1,500, and 1,600 Hz; 50 ms; 1,000 stimuli in total) were presented with an equal probability of 10%. The stimulus onset asynchrony was 503 ms. Stimulus presentation was controlled by MATLAB (MathWorks Inc., Natick, MA, USA) using the Psychophysics Toolbox extensions (39, 40). Tones were presented through two audio speakers (Fostex, Japan) with an average intensity of 70 dB SPL around the animal’s ear.

### ECoG Recordings

#### General Settings

ECoG signals were recorded at a sampling rate of 1 kHz using a Grapevine NIP (Ripple Neuro, Salt Lake City, UT). In each recording session, only an auditory stimuli (AD or AF) was used. During the recordings, the monkeys were placed in a sphinx position in a custom bed in a dimly lit, electrically shielded and sound-attenuated chamber with their head fixed. For awake recordings, 5, 4, and 2 sessions were conducted on monkey S, R, and J on separate days. For monkeys M and O, one recording session was conducted. Different numbers of recording sessions were caused by experimental schedules of each animal used in different research projects.

#### Recordings From Marmosets With Ketamine Administration

We conducted experiments with ketamine administration on three monkeys (J, M, and O). On each experimental day, a recording session with awake marmosets was first conducted. Next, an anesthetic dose of ketamine (30 mg/kg i.m.) was injected 5 min after an atropine (0.08 mg/kg i.m.) injection. Ten minutes after the injection, ECoG recordings were repeatedly conducted at approximately 30-min intervals for up to 3 h. For monkey J, the AD and AF experiments were conducted on two different days. For monkeys M and O, the AD and AF experiments were conducted on one experimental day. We discarded the data from one experimental day for monkeys J and M since they struggled during the sessions. In the following analysis, we focused on 10, 30, and 150 min following administration (K10, K30, and K150) since increased muscle tone [a common side effect of ketamine (41)] causes artifacts on high-gamma activity during 60–120 min following administration.

### Data Analysis

#### Preprocessing

**Figure 1:**
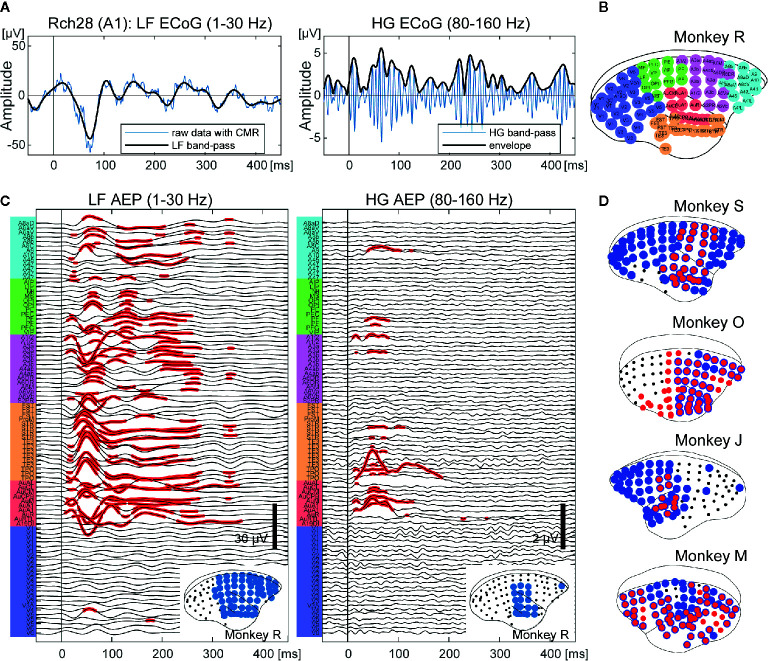
Cortex-wide auditory evoked responses. **(A)** An example of an ECoG signal of a single trial from the primary auditory area of monkey R. Left: an example of a low frequency (1–30 Hz) ECoG (LF). Right: an example of a high frequency (80–160 Hz) ECoG (HG). The vertical bars indicate the onset of sound stimuli. **(B)** The locations of electrodes and putative cortical areas. **(C)** Auditory evoked potentials of monkey R. LF (left) and HG (right) ECoGs are aligned to sound onset and averaged. The x-axis represents the times and the y-axis represents the labels of the ECoG electrodes in **(B)**. The red dots indicate the electrodes and times that show significant responses (adjusted p < 0.00001). The bottom insets show the cortical map for auditory stimuli. The black dots represent the electrode positions. The blue circles represent the electrodes that showed significant responses. **(D)** Cortical maps for auditory information processing from the other four monkeys. The black dots represent the electrode positions. The blue and red circles represent the electrodes that showed significant responses in LF and HG, respectively. Individual AEPs are shown in **Supplementary Figure 1**.

Electrophysiological data were analyzed using custom scripts for MATLAB. For signal pre-processing, signals were re-referenced using a common median reference (CMR) montage, and band-pass filtered at 1–30 Hz (LF) and 80–160 Hz (HG). We calculated the envelope of the HG frequency band using the Hilbert transform (**Figure 1A**). We assumed that the LF mainly reflected a summation of post-synaptic potentials, while HG was related to mean firing activity (14, 42, 43). We segmented datasets from −100 to 450 ms relative to the onsets of the tones. To remove segments that contained outliers, we calculated a standard deviation (SD_i_, i = 1–nT, in which nT is a number of trials) for each segment and for all segments (SD_all_) and removed segments with an SD_i_ above 3SD_all_. After excluding outlier, we applied a baseline correction by subtracting the mean of the 100 ms period prior to stimulus onset. The ERPs were then calculated for each channel.

#### Auditory Evoked Responses (AERs)

To distinguish spontaneous signals in the baseline period and responses to auditory stimuli, we conducted t-tests at each time point 0 to 450 ms after stimulus onset for every electrode. All p-values from each monkey and each recording session were false discovery rate (FDR)-adjusted (44). The significance level was set to 0.00001 unless otherwise specified.

#### Feature Specific Responses

To examine selective responses for auditory features, frequencies, and durations, we first conducted a one-way analysis of variance (ANOVA) on frequencies or on durations at each time point at 0 to 450 ms for each electrode. All p-values from each monkey and each recording session were FDR-adjusted. The significance level was set to 0.00001 unless otherwise specified. For frequency, we further determined the best frequency for each electrode. We calculated the means of responses for each frequency over statistically significant time points, and defined the frequency showing the maximum value of the means as the best frequency (BF).

#### Ketamine Effects

To evaluate the effects of ketamine on AERs, we quantified the effects of ketamine administrations as differences in the amplitudes and latencies of the peaks of LF and mean responses of HG. For LF, we identified the first and second peaks of the AERs of electrodes at the significant time points, and obtained their amplitudes and latencies. To statistically compare those values of awake with ketamine conditions, we conducted a two-sample t-test within each electrode group based on putative cortical areas. For visualization purposes, we then normalized the amplitudes by dividing by the value of the awake condition, and calculated the differences in the latencies by subtracting the values obtained from awake conditions. For HG, we calculated the mean values of early (0–30 ms) and late (30–100 ms) responses following tone onset. To investigate the effects of ketamine on the observed responses, we calculated the z-score across conditions (i.e., Awake, K10, K30, K150) at each electrode, then subtracted the z-score of awake conditions from that of ketamine conditions. We determined the significant effects as less than −1.96 or as more than 1.96.

To evaluate the effects of ketamine on auditory feature processing, we quantified the changes in BFs and in onset/offset response ratios of selected electrodes, which showed both significant frequency preferences and offset responses in awake conditions. To calculate onset/offset response ratios, we calculated the means of early (0–200) and late (200–400) HG responses for tones with 200 and 225 ms durations, since neural activity during the early time windows for those two tones do not include offset response. Then we obtained onset/offset response ratios by dividing early HG responses by late HG responses.

The numbers of electrodes showing significant responses following ketamine administration were compared with those of the awake condition using a two-sample t-test with a significance level set to 0.05.

## Results

### Cortex-Wide Auditory Responses in Awake Marmosets

We collected whole-cortical ECoGs from five awake marmosets on presentation of auditory stimuli of different frequencies and durations. First, we calculated event related responses (ERRs) averaged over all AF stimuli for both LF and HG, and then compared the amplitudes at each time point from 0 to 450 ms following stimulus onset with the baseline (100 to 0 prior to onset) activity of each electrode. **Figure 1C** shows the AERs of LF and HG from monkey R. For LF, significant auditory responses in LF occurred in a large part of the cortex, including frontal and parietal cortices, as well as in temporal cortex (62.5% of the electrodes of monkey R). HG responses appeared in more limited areas (12.5%). Similar tendencies were observed in all animals (**Figure 1D** and **Supplementary Figure S2**).

**Figure 2:**
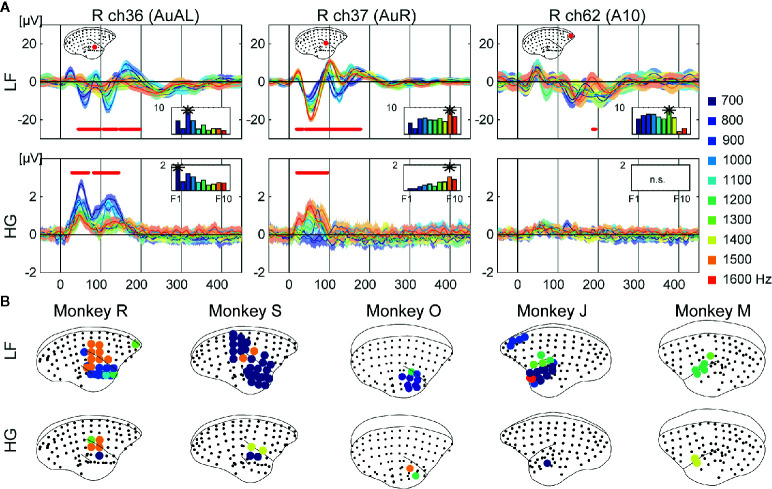
Frequency preference. **(A)** Examples of representative ECoG responses to each frequency at selective electrodes of monkey R. Mean responses to each 10 frequencies are represented. The shaded areas correspond to 95% confidence intervals. The red dots represent time points which showed significant different responses to frequencies. The bar plot represents the mean responses to each frequency over time which were significant. The best frequencies of the electrodes are represented as asterisks. **(B)** Cortical maps of the tonotopy of LF (top insets) and HG (bottom insets). The circles indicate electrodes which show statistical significance, with colors representing the best frequency.

We next examined selective responses to auditory features, frequencies, and durations. For frequency, we conducted a one-way ANOVA on frequencies at each time point from 0 to 450 ms for each electrode. The electrodes around the temporal sulcus showed a tonotopic spatial distributions of electrodes, while other significant electrodes did not show any significant difference in frequencies. For example, in monkey J, electrode 36 showed larger responses to low frequency tones, while electrode 37 showed larger responses for high frequencies (**Figure 2A**). Furthermore, the frequencies yielding the largest responses varied over time. We calculated the means of responses for each frequency over statistically significant time points, and defined the frequency showing the maximum value as the best frequency (BF). The BFs of electrodes in dorsal auditory cortices tended to be high frequencies for both LF and HG, and the significant electrodes of HG (3.3 ± 1.5%; mean ± SD of all monkeys) showed more limited distributions than those of LF (18.6 ± 9.7%) (**Figure 2B**).

**Figure 3:**
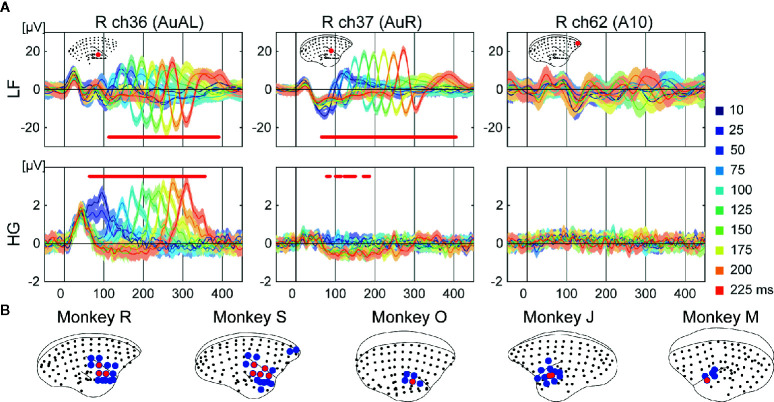
Tone offset responses. **(A)** Examples of representative ECoG responses to each duration on selective electrodes of monkey R. Mean responses to each 10 duration are represented. The shaded areas correspond to the 95% confidence intervals. The red dots represent the time points which showed significantly different responses to durations. **(B)** Cortical maps of the tonotopy of LF (blue) and HG (red).

We conducted a one-way ANOVA on durations at each time point from 0 to 450 ms following tone onset for each electrode. The electrodes around the temporal sulcus showed sound offset responses (**Figure 3**). Unlike frequency-related responses, sound offset responses were found in the same electrodes. The significant electrodes of HG (2.6 ± 1.7%) showed more limited distributions than those of LF (11.3 ± 2.6%) (**Figure 3B**).

### Cortex-Wide Auditory Responses in Marmosets With Ketamine Administration

**Figure 4:**
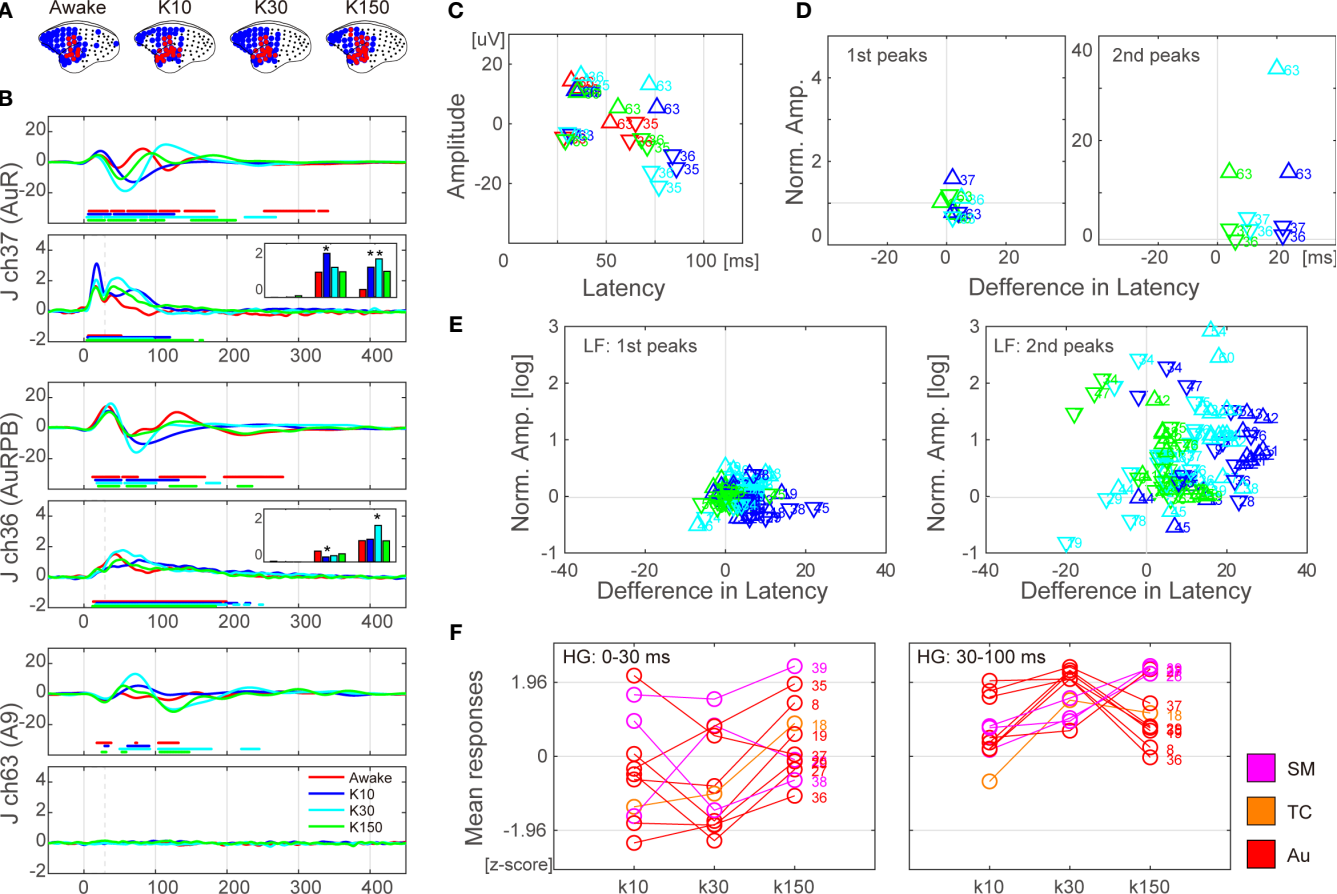
The effects of ketamine on auditory evoked neural responses over time following ketamine administration in monkey J. **(A)** Changes in cortical maps for auditory information processing of LF (blue) and HG (red). Data were acquired form monkey J. **(B)** Representative waveforms from electrodes in auditory (ch37 and ch36) and prefrontal (ch63) cortices. The top and bottom insets show LF and HG, respectively. The line color indicates the conditions of the recordings, and the dots represent significant time points. **(C)** The amplitudes and latencies of the 1st and 2nd peaks of LF of the selected electrodes. The marker color indicates the conditions of the recordings. The triangles represent positive peaks, and the inverted triangles represent negative peaks. **(D)** Normalized amplitudes and latencies of the 1st (left) and 2nd (right) peaks of the selected electrodes. **(E)** Normalized amplitudes and latencies of the 1st (left) and 2nd (right) peaks of all electrodes. The amplitudes are log scaled. **(F)** Changes in responses of HG at 0–30 ms (left) and 30–100 ms (right) after the sound onset. The bar plots in **(B)** show these values of the corresponding electrodes. Asterisks in the bar plots indicate significant (>2) changes.

We examined the effects of ketamine administration on cortex-wide auditory information processing in three marmosets at approximately 10 (K10), 30 (K30), and 150 (K150) min following administration. AERs were observed in temporal, parietal, and frontal areas (**Figure 4A** and **Supplementary Figure S3A**). The number of electrodes showing significant AERs did not change significantly across subjects following ketamine administration. **Figure 4B** and **Supplementary Figure S3B** show the signal changes in representative electrodes in auditory and frontal areas. We quantified the effects of ketamine administration as the differences in amplitudes and latencies of the peaks of LF and mean responses of HG. For LF, we identified the first and second peaks of the AERs of electrodes at significant time points and obtained their amplitudes and latencies. **Figures 4C** and **S3C** show the amplitudes and latencies of the representative electrodes in **Figures 4B** and **S3B**. We next calculated the differences from these values obtained from awake conditions (**Supplementary Table S2**, **Figures 4D, E**, and **Supplementary Figures S3D, E**). The amplitudes and the latencies of the first peaks remained relatively unchanged, while most of the amplitudes and latencies of the second peaks increased after 10 and 30 ms following administration. Moreover, the amplitudes of the first peaks did not change much in all monkeys, while the PFC latencies increased after 10 and 30 ms following administration. In the second peaks, both amplitudes and latencies of PFC were significantly increased after 10 and 30 ms following administration (**Supplementary Table S2**). For HG, we calculated the mean values of early (0–30 ms) and late (30–100 ms) responses. Most of the electrodes showed increased HG responses following ketamine administration (**Figure 4F** and **Supplementary Figure S3**). Only early responses in the auditory areas of monkey J decreased 10–30 min following ketamine administration.

**Figure 5:**
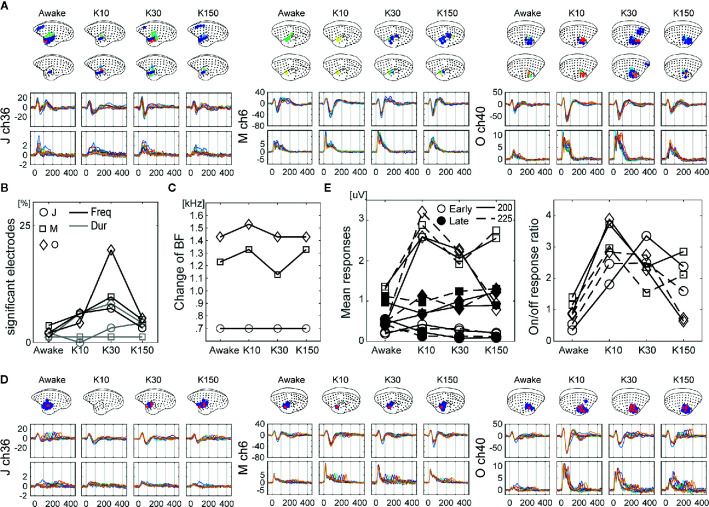
The effects of ketamine on auditory feature processing over time following ketamine administration. **(A)** Changes in cortical maps for frequency preference of LF (top brain insets) and HG (bottom brain insets). The LF and HG signals of representative electrodes for LF and HG are shown. The dot and line colors represent each frequency. **(B)** Changes in the number of significant electrodes in terms of frequency preferences (black lines), and offset responses (gray lines). Data from monkey J (circle), M (square), and O (cross). Changes in BF **(C)**. **(D)** Changes in cortical maps for offset responses of LF (blue) and HG (red) (top). The LF (middle) and HG (bottom) signals of representative electrodes for LF and HG are shown. The dot and line colors indicate each duration. **(E)** Means of onset (0–200 ms; filled markers) and offset (200–400 ms; open markers) responses for the tones with 200 (solid lines) and 225 ms (dashed lines) (left). On/off response ratio (right).

We next examined the effects on auditory feature processing, and observed changes in all three monkeys. Regarding frequency preference, we observed a significant increase in the numbers of electrodes, which showed a frequency preference for HG in K10 (**Figures 5A, B** black lines). For HG in K30 and K150, the numbers of significant electrodes also increased but not significantly (paired t-test *p* = 0.07 and 0.10, respectively). Thereafter, we examined the frequency preference of selected electrodes. The preference for higher or lower tones was not strongly affected by ketamine. Changes in BF were not observed in monkey J, while few (<100 Hz) changes were observed in monkeys M and O (**Figure 5C**). For sound offset responses, no significant changes were observed in the number of electrodes which showed sound offset responses (**Figures 5D, B** gray lines), while some of the monkeys showed tendencies similar to the frequency preferences. The number of significant electrodes of HG at 10–150 min and 30–150 min following administration in monkeys M and J, respectively, were increased. Furthermore, we calculated the means of early (0–200) and late (200–400) HG responses for tones of 200- and 225-ms duration (**Figure 5E** left), and then calculated the onset/offset response ratios. Results demonstrated that onset/offset response ratios increased significantly over time following ketamine administration (**Figure 5E** right). The onset/offset balance 150 ms following ketamine administration of monkey O returned to its awake value, though the mean offset responses remained larger than the awake responses. Finally, to confirm that the different effects of ketamine on frequency and duration are not contingent on the different experimental days, we compared responses to a 50-ms tone at 1,000 Hz recorded in AF and AD experiments. Even though AF and AD experiments were conducted on different days, we observed similar wave forms (**Supplementary Figure S4**).

## Discussion

To the best of our knowledge, this study is the first to demonstrate the effect of ketamine in cortex-wide auditory evoked responses in nonhuman primates. We first recorded ECoGs from awake monkeys on presentation of auditory stimuli of different frequencies or durations. We observed AERs across the cortex, including in frontal, parietal, and temporal areas, while feature specific responses were obtained around the temporal sulcus. Next, we examined the effects of ketamine on cortical auditory information processing. We conducted ECoG recordings from monkeys between 10 and 180 min following ketamine administration, and observed significant changes in stimulus feature-specific responses. The number of electrodes which showed frequency preferences or offset responses changed following ketamine administration, while that of AERs was not largely influenced. However, the frequency preference of a selected electrode was not heavily modulated by ketamine administration over time following administration, while the imbalances in onset and offset persisted over the course of the 150 min following ketamine administration in all three monkeys.

### Cortex-Wide Auditory Responses in Awake Marmosets

We conducted ECoG recordings from awake marmosets on presentation of auditory stimuli, and found that auditory evoked potentials were observed across the cortex including in frontal, parietal, and temporal areas (**Figure 1**). Furthermore, feature-specific responses were obtained around the temporal sulcus. Regarding frequency, we observed a tonotopic organization whereby the BFs of electrodes in the dorsal part of the auditory cortices tended to be of high frequencies both in LF and HG (**Figure 2**). These results are consistent with those of previous studies on tonotopic representations in the primary auditory area of marmosets (45–47). However, the frequency range of the current study was limited to 600–1,700 Hz, while frequency ranges in previous studies varied from 125 to 38,100 Hz. Thus, more broad frequency ranges are required to construct a more complete tonotopic organization. As per duration, we found sound offset responses in the small number of electrodes which showed onset responses (**Figure 3**), although previous studies have reported that sound offset responses are found throughout the auditory cortex and subcortical structures. This may be due to the fact that we varied the durations of tones by only 1,000 Hz, and that offset responses also have frequency preferences (48).

### Cortex-Wide Auditory Responses in Marmosets With Ketamine Administration

Next, we examined the effects of an anesthetic dose of ketamine on auditory responses, and observed a significant increase in the auditory evoked responses and latencies for both LF and HG signals from many electrodes. These observations are inconsistent with those of previous studies. Schwender, Klasing (31) reported that there was no change in the latencies or amplitudes of the mid-latency peaks of AEPs after induction of general anesthesia with ketamine according to human electroencephalography (EEG) data. Umbricht, Schmid (28) also reported that there were significant changes in N1 amplitude, though its latencies were not significant. Furthermore, Javitt and colleagues reported that PCP administration inhibits auditory N1 responses in the primary auditory area of macaque monkeys as shown using cranial electrodes on Cz (49) or depth electrodes on A1 (17). There are several possible reasons for these varied reports with regard to the effects of ketamine. First, these three studies used different doses and administration routes, which are known to strongly influence the effects of ketamine (50). Second, the nature of the sound stimuli was another modulating factor. The amplitude of N1 depends on the inter stimulus interval (ISI) of the tone sequence, and PCP enhances monkey N1 responses to tone sequences with short ISIs, while it decreases monkey N1 responses to tone sequences with long ISIs (49). Thus, the effects of ketamine may vary depending on the sound stimuli. Third, the timing of the recordings relative to the administration of ketamine also had an influence. In our study, increased amplitudes diminished over time. Thus, if we were to conduct observations for longer periods of time, there is a possibility that there may be a time zone during which auditory responses are less strong than those of awake conditions. Despite these inconsistencies, current results were consistent between all three subjects and appeared not to contradict the fact that ketamine induces auditory hallucinations.

### Frequency- and Duration-Related Responses With Ketamine Administration

Finally, we investigated the effects of ketamine on feature-specific responses. The numbers of electrodes that showed frequency preferences or offset responses were altered following the administration of ketamine. However, the BF of a selected electrode was not modulated much by ketamine administration over time following the administration, while the imbalances in onset and offset persisted over the course of 150 min following the administration of ketamine in all three monkeys. These results suggest that ketamine affects the ability to distinguish between sound frequency and duration in different ways. Despite the difficulty in specifying the precise molecular mechanism of the observed ketamine effects, different influences of NMDA receptors blockade on sound duration and frequency may be able to explain the alterations in duration and frequency MMN at different stages of schizophrenia. Friedman, Sehatpour (51) reported that the deficits in duration MMN processing may be specifically related to impaired premorbid function, whereas deficits in MMN to frequency deviants may continue to worsen even during early disease stages (52, 53). Furthermore, a similar imbalance in the onset and offset responses in K10 has been reported in an electrophysiological study on the primary auditory area of aged monkeys (54). This study also showed that the increase in firing rates of auditory cortex neurons in aged monkeys was significantly higher than that in young monkeys, a result consistent with those of previous studies (55–57). The authors argued that this result was caused by weakened inhibition in the ascending auditory pathway. In addition to these experiments on aged monkeys, Scholl, Gao (58) reported that sound offset responses of rat auditory cortex are not only products of local inhibitory circuits but also of the auditory ascending pathway. Therefore, ketamine may suppress the auditory ascending pathway at least immediately following administration. Kopp-Scheinpflug, Sinclair (59) argued that specific abnormalities in sound offset responses may, rather than reflect problems in tone-detection thresholds, reflect impairments in temporal processing and the ability to rapidly perceive varying signals, such as auditory scene analysis and speech perception. As such, understanding the cellular and circuit origins of sound offset responses in the auditory brain may be key to advancing the treatment of auditory temporal processing deficits in aging and disease.

In this study, we focused on high-gamma band ECoG power as well as low-frequency ECoG. Aberrant gamma-band oscillations have been ubiquitously observed in schizophrenia and in animal models of ketamine administrations [e.g., (20, 60)]. Several studies have reported excessive gamma oscillatory activity in schizophrenia (61) and in animal with ketamine administrations (14). Skoblenick, Womelsdorf (14) argued that the increased gamma-band activity may reduce the signal-to-noise ratio of task related neurons. It may be that the increased significant electrodes observed in the auditory feature specific responses may be another manifestation of an increase in background noise and dysfunctions of auditory feature discriminations. Further investigation is required to confirm this hypothesis.

There are some limitations to this study. First, the variations in the frequencies and durations of sound stimuli were limited in scope. These current results need to be confirmed using a broader range of different parameters since onset/offset balance is affected by stimulus intensity and frequency. Second, the study was based on a small number of subjects. In this study, three monkeys showed several different results in response to the administration of ketamine. This was to be expected since psychotomimetic effects are reported in only 43% of human subjects (62). It is essential to acquire and accumulate larger dataset from animal models in order to compare these results with reports from human subjects. Third, we examined data from limited time frames. During 60–120 min following ketamine administration, increased muscle tone was observed and caused artifacts on high-gamma activity.

In conclusion, whole cortex ECoG allows us to investigate cortex-wide information processing. Recent results indicate that frontal and parietal areas are involved in auditory information processing, as are temporal areas. However, stimulus feature-specific responses were primarily observed around the temporal sulcus. The administration of ketamine affected these stimulus-feature specific processes. Future research on the NMDA sensitivity of cortex-wide auditory information processing may provide a new perspective which will help to develop NHP models to study psychiatric disorders.

## Data Availability Statement

The raw data supporting the conclusions of this article are available in online repositories, Brain/MINDS Data Portal (Data ID: 4924).

## Ethics Statement

The animal study was reviewed and approved by RIKEN Ethical Committee (No. H28-2-221(3)).

## Author Contributions

MK designed and conducted the experiments, and analyzed the data. NI supervised the project. All authors contributed to the article and approved the submitted version.

## Funding

This study was supported by the Brain Mapping by Integrated Neurotechnologies for Disease Studies (Brain/MINDS) project of the Japan Agency for Medical Research and Development (AMED) (JP20dm0207069, JP20dm0207001) and by the Japan Society for the Promotion of Science (JSPS) KAKENHI (JP19H04993, JP17H06034).

## Conflict of Interest

The authors declare that the research was conducted in the absence of any commercial or financial relationships that could be construed as potential conflict of interest.
