# Supplementary material for: Effects of Ketamine Administration on Auditory Information Processing in the Neocortex of Nonhuman Primates
Source: Front Psychiatry. 2020 Aug 19;11:826. doi: 10.3389/fpsyt.2020.00826 (PMC7466740; doi:10.3389/fpsyt.2020.00826)
Supplement: Supplementary file 1 [file Image_1.pdf]

Supplementary Material

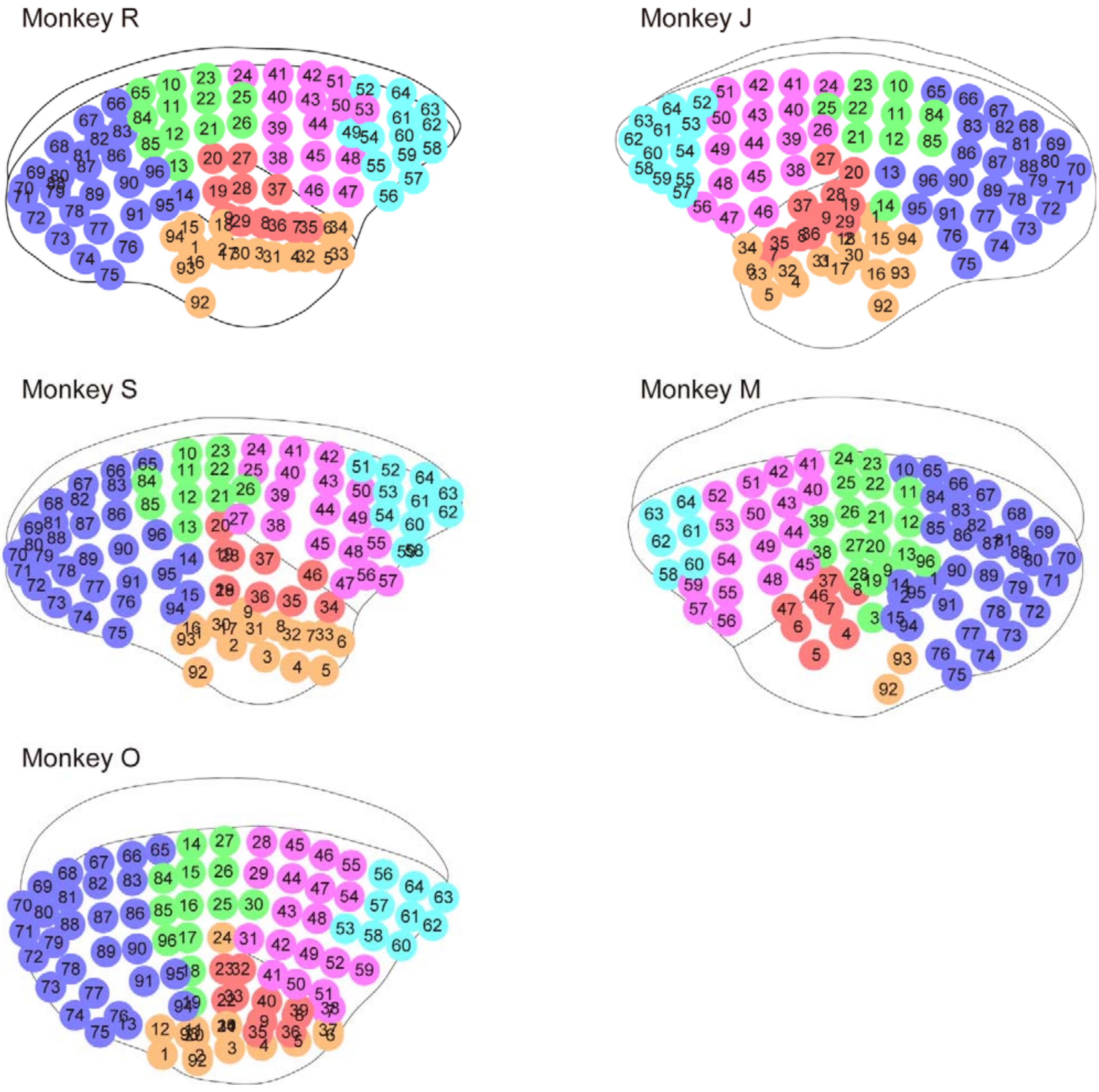

Supplementary Figure S1. The channel number and location of all the electrodes in all monkeys. The putative cortical areas for every electrode are provided in Supplementary Table S1. Based on the putative cortical areas, electrodes were divided into six groups, corresponding to the prefrontal

(cyan), sensorimotor (magenta), posterior parietal (green), visual (blue), temporal (orange), and auditory (red) areas.

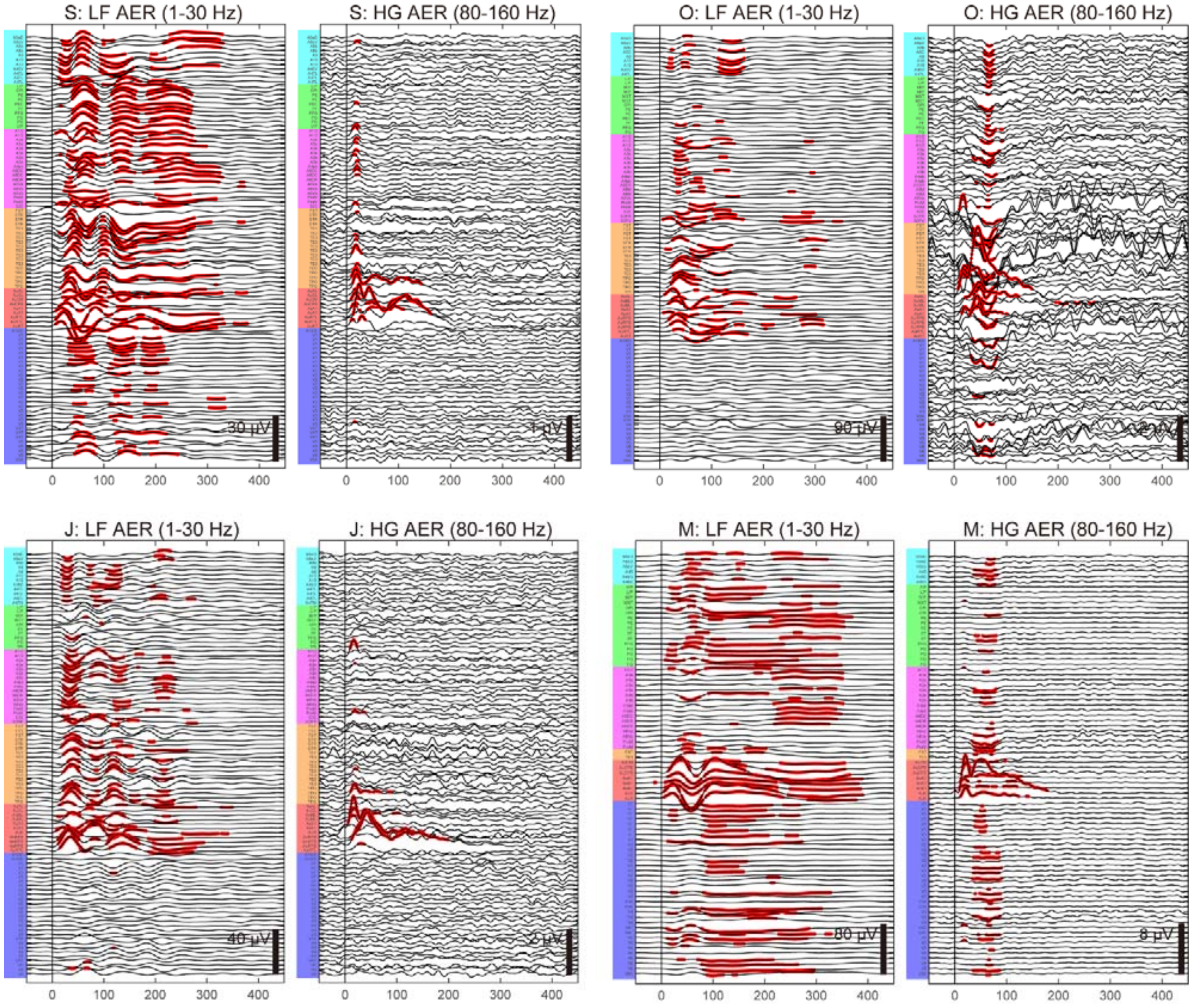

Supplementary Figure S2. Individual AERs. LF (left) and HG (right) ECoGs are aligned to sound onset and averaged. The X-axis represents times and the Y-axis represents the labels of the ECoG electrodes. The red dots indicate the electrodes and the times that showed significant responses.

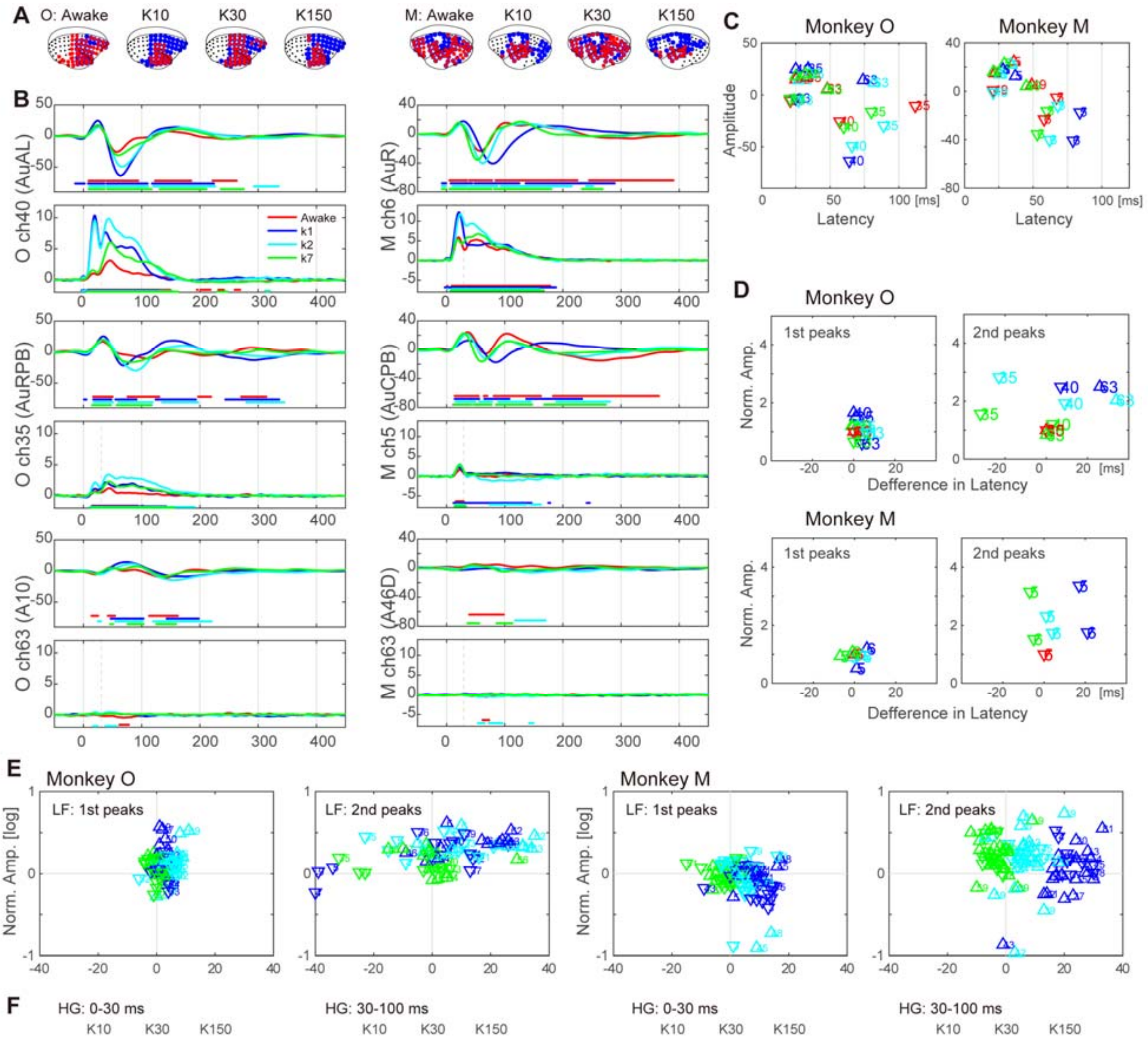

Supplementary Figure S3. Ketamine effects on auditory evoked neural responses over time following ketamine administration in monkeys M and O. (A) Changes in cortical maps for auditory information processing of LF (blue) and HG (red). (B) Representative waveforms from electrodes in auditory and prefrontal cortices. The top and bottom insets show LF and HG, respectively. The line colour indicates the conditions of recordings, and the dots represent significant time points. (C) The amplitudes and latencies of the 1st and 2nd peaks of LF of the selected electrodes. The marker colour represents the conditions of recordings. The triangles represent positive peaks, and the inverted triangles represent negative peaks. (D) Normalized amplitudes and latencies of the 1st (left) and 2nd (right) peaks of the selected electrodes. (E) Normalized amplitudes and latencies of the 1st (left) and 2nd (right) peaks of all electrodes. The amplitudes are log scaled. (F) Changes in responses of HG at 0-30 ms (left) and 30-100 ms (right) after the sound onset.

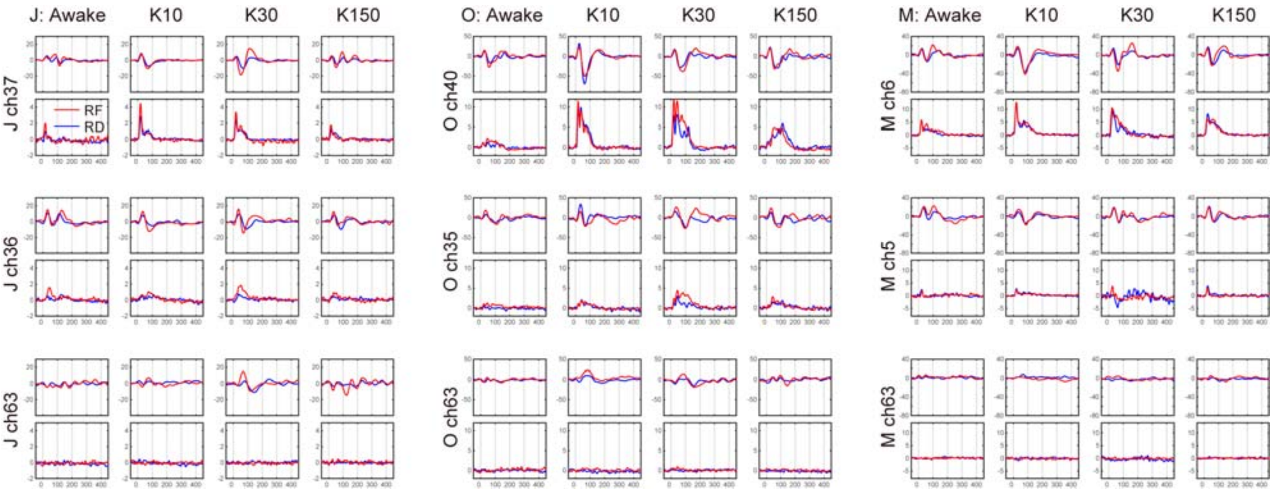

Supplementary Figure S4. LF and HG responses for a 50-ms tone at 1000 Hz during AF and AD experiments.
