# Supplementary material for: Effects of Ketamine Administration on Auditory Information Processing in the Neocortex of Nonhuman Primates
Source: Front Psychiatry. 2020 Aug 19;11:826. doi: 10.3389/fpsyt.2020.00826 (PMC7466740; doi:10.3389/fpsyt.2020.00826)
Supplement: Supplementary file 2 [file Table_1.pdf]

Monkey R

ch#	Name of cortical areas	Abbrebiations
1	'Fundus of superior temporal sulcus area of cortex'	'FST'
2	'Temporal area TE3'	'TE3'
3	'Temporo-parieto-occipital association area (superior temporal polysensory cortex)'	'TPO'
4	'Superior temporal rostral area'	'STR'
5	'Superior temporal rostral area'	'STR'
6	'Superior temporal rostral area'	'STR'
7	'Auditory cortex; rostrotemporal lateral area'	'AuRTL'
8	'Auditory cortex; anterolateral area'	'AuAL'
9	'Auditory cortex; middle lateral area'	'AuML'
10	'Parietal area PE; caudal part'	'PEC'
11	'Ventral intraparietal area of cortex'	'VIP'
12	'Occipito-parietal transitional area of cortex'	'OPt'
13	'Medial superior temporal area of cortex'	'MST'
14	'Visual area 5 (middle temporal area)'	'V5'
15	'Fundus of superior temporal sulcus area of cortex'	'FST'
16	'Temporal area TE3'	'TE3'
17	'Temporal area TE3'	'TE3'
18	'Temporo-parieto-occipital association area (superior temporal polysensory cortex)'	'TPO'
19	'Auditory cortex; caudolateral area'	'AuCL'
20	'Auditory cortex; caudomedial area'	'AuCM'
21	'Parietal area PFG'	'PFG'
22	'Anterior intraparietal area of cortex'	'AIP'
23	'Parietal area PE'	'PE'
24	'Areas 1 and 2 of cortex'	'A1/2'
25	'Parietal area PF'	'PF'
26	'Parietal area PF'	'PF'
27	'Auditory cortex; primary area'	'AuA1'
28	'Auditory cortex; primary area'	'AuA1'
29	'Auditory cortex; caudal parabelt area'	'AuCPB'
30	'Temporal area TE3'	'TE3'
31	'Temporo-parieto-occipital association area (superior temporal polysensory cortex)'	'TPO'
32	'Superior temporal rostral area'	'STR'
33	'Superior temporal rostral area'	'STR'
34	'Proisocortical motor region (precentral opercular cortex)'	'ProM'
35	'Auditory cortex; rostrotemporal lateral area'	'AuRTL'
36	'Auditory cortex; anterolateral area'	'AuAL'
37	'Auditory cortex; rostral area'	'AuR'
38	'Areas 1 and 2 of cortex'	'A1/2'
39	'Area 3b of cortex (primary somatosensory)'	'A3b'
40	'Area 3b of cortex (primary somatosensory)'	'A3b'
41	'Area 3a of cortex (primary somatosensory)'	'A3a'
42	'Area 4 of cortex; parts a and b (primary motor)'	'A4ab'
43	'Area 4 of cortex; parts a and b (primary motor)'	'A4ab'
44	'Area 3a of cortex (primary somatosensory)'	'A3a'
45	'Area 3b of cortex (primary somatosensory)'	'A3b'
46	'Secondary somatosensory cortex; parietal rostral area'	'S2PR'
47	'Area 6 of cortex; ventral; part b'	'A6Vb'
48	'Area 6 of cortex; ventral; part a'	'A6Va'
49	'Area 8 of cortex; caudal part'	'A8C'

50	'Area 6 of cortex; dorsorostral part'	'A6DR'
51	'Area 6 of cortex; medial (supplementary motor) part'	'A6M'
52	'Area 8b of cortex'	'A8b'
53	'Area 6 of cortex; dorsorostral part'	'A6DR'
54	'Area 8a of cortex; ventral part'	'A8aV'
55	'Area 45 of cortex'	'A45'
56	'Area 47 (old 12) of cortex; lateral part'	'A47L'
57	'Area 47 (old 12) of cortex; lateral part'	'A47L'
58	'Area 47 (old 12) of cortex; lateral part'	'A47L'
59	'Area 47 (old 12) of cortex; lateral part'	'A47L'
60	'Area 8a of cortex; ventral part'	'A8aV'
61	'Area 8a of cortex; dorsal part'	'A8aD'
62	'Area 10 of cortex'	'A10'
63	'Area 9 of cortex'	'A9'
64	'Area 8b of cortex'	'A8b'
65	'Medial intraparietal area of cortex'	'MIP'
66	'Visual area 6 (dorsomedial area)'	'V6'
67	'Visual area 6 (dorsomedial area)'	'V6'
68	'Visual area 2'	'V2'
69	'Visual area 1'	'V1'
70	'Visual area 1'	'V1'
71	'Visual area 1'	'V1'
72	'Visual area 1'	'V1'
73	'Visual area 1'	'V1'
74	'Visual area 3 (ventrolateral posterior area)'	'V3'
75	'Visual area 3 (ventrolateral posterior area)'	'V3'
76	'Visual area 4 (ventrolatereral anterior area)'	'V4'
77	'Visual area 3 (ventrolateral posterior area)'	'V3'
78	'Visual area 2'	'V2'
79	'Visual area 1'	'V1'
80	'Visual area 1'	'V1'
81	'Visual area 2'	'V2'
82	'Visual area 2'	'V2'
83	'Visual area 3A (dorsoanterior area)'	'V3A'
84	'Lateral intrapartietal area of cortex'	'LIP'
85	'Occipito-parietal transitional area of cortex'	'OPt'
86	'Visual area 3 (ventrolateral posterior area)'	'V3'
87	'Area 19 of cortex; dorsointermediate part'	'A19DI'
88	'Visual area 2'	'V2'
89	'Visual area 2'	'V2'
90	'Visual area 3 (ventrolateral posterior area)'	'V3'
91	'Visual area 4 (ventrolatereral anterior area)'	'V4'
92	'Temporal area TE3'	'TE3'
93	'Temporal area TE3'	'TE3'
94	'Fundus of superior temporal sulcus area of cortex'	'FST'
95	'Visual area 5 (middle temporal area)'	'V5'
96	'Visual area 5 (middle temporal area)'	'V5'

Monkey S

ch#	Name of cortical areas	Abbrebiations
1	'Temporal area TE3'	'TE3'
2	'Temporal area TE3'	'TE3'
3	'Temporal area TE3'	'TE3'
4	'Temporal area TE2'	'TE2'
5	'Temporal area TE1'	'TE1'
6	'Superior temporal rostral area'	'STR'
7	'Superior temporal rostral area'	'STR'
8	'Temporo-parieto-occipital association area (superior temporal polysensory cortex)'	'TPO'
9	'Temporo-parieto-occipital association area (superior temporal polysensory cortex)'	'TPO'
10	'Parietal area PE; caudal part'	'PEC'
11	'Ventral intraparietal area of cortex'	'VIP'
12	'Parietal area PG'	'PG'
13	'Parietal area PG'	'PG'
14	'Visual area 5 (middle temporal area)'	'V5'
15	'Visual area 4; transitional part'	'V4T'
16	'Temporal area TE3'	'TE3'
17	'Temporal area TE3'	'TE3'
18	'Temporo-parieto-occipital association area (superior temporal polysensory cortex)'	'TPO'
19	'Auditory cortex; caudolateral area'	'AuCL'
20	'Auditory cortex; caudomedial area'	'AuCM'
21	'Parietal area PFG'	'PFG'
22	'Parietal area PE'	'PE'
23	'Parietal area PE'	'PE'
24	'Area 3a of cortex (primary somatosensory)'	'A3a'
25	'Areas 1 and 2 of cortex'	'A1/2'
26	'Parietal area PF'	'PF'
27	'Secondary somatosensory cortex; external part'	'S2E'
28	'Auditory cortex; primary area'	'AuA1'
29	'Auditory cortex; caudal parabelt area'	'AuCPB'
30	'Fundus of superior temporal sulcus area of cortex'	'FST'
31	'Temporal area TE3'	'TE3'
32	'Temporo-parieto-occipital association area (superior temporal polysensory cortex)'	'TPO'
33	'Superior temporal rostral area'	'STR'
34	'Auditory cortex; rostrotemporal lateral area'	'AuRTL'
35	'Auditory cortex; rostrotemporal lateral area'	'AuRTL'
36	'Auditory cortex; anterolateral area'	'AuAL'
37	'Auditory cortex; primary area'	'AuA1'
38	'Areas 1 and 2 of cortex'	'A1/2'
39	'Area 3b of cortex (primary somatosensory)'	'A3b'
40	'Area 3a of cortex (primary somatosensory)'	'A3a'
41	'Area 4 of cortex; parts a and b (primary motor)'	'A4ab'
42	'Area 6 of cortex; dorsocaudal part'	'A6DC'
43	'Area 6 of cortex; dorsocaudal part'	'A6DC'
44	'Area 3a of cortex (primary somatosensory)'	'A3a'
45	'Area 3b of cortex (primary somatosensory)'	'A3b'
46	'Auditory cortex; rostrotemporal part'	'AuRT'
47	'Proisocortical motor region (precentral opercular cortex)'	'ProM'
48	'Area 3a of cortex (primary somatosensory)'	'A3a'
49	'Area 6 of cortex; ventral; part a'	'A6Va'

50	'Area 6 of cortex; dorsorostral part'	'A6DR'
51	'Area 8b of cortex'	'A8b'
52	'Area 8b of cortex'	'A8b'
53	'Area 8a of cortex; dorsal part'	'A8aD'
54	'Area 8a of cortex; ventral part'	'A8aV'
55	'Area 6 of cortex; ventral; part b'	'A6Vb'
56	'Area 6 of cortex; ventral; part b'	'A6Vb'
57	'Proisocortical motor region (precentral opercular cortex)'	'ProM'
58	'Area 47 (old 12) of cortex; lateral part'	'A47L'
59	'Area 47 (old 12) of cortex; lateral part'	'A47L'
60	'Area 47 (old 12) of cortex; lateral part'	'A47L'
61	'Area 46 of cortex; ventral part'	'A46V'
62	'Area 10 of cortex'	'A10'
63	'Area 10 of cortex'	'A10'
64	'Area 9 of cortex'	'A9'
65	'Visual area 6A (posterior parietal medial area)'	'V6A'
66	'Visual area 6 (dorsomedial area)'	'V6'
67	'Visual area 2'	'V2'
68	'Visual area 2'	'V2'
69	'Visual area 1'	'V1'
70	'Visual area 1'	'V1'
71	'Visual area 1'	'V1'
72	'Visual area 1'	'V1'
73	'Visual area 1'	'V1'
74	'Visual area 2'	'V2'
75	'Visual area 3 (ventrolateral posterior area)'	'V3'
76	'Visual area 3 (ventrolateral posterior area)'	'V3'
77	'Visual area 2'	'V2'
78	'Visual area 2'	'V2'
79	'Visual area 1'	'V1'
80	'Visual area 1'	'V1'
81	'Visual area 2'	'V2'
82	'Visual area 2'	'V2'
83	'Visual area 6 (dorsomedial area)'	'V6'
84	'Lateral intrapartietal area of cortex'	'LIP'
85	'Occipito-parietal transitional area of cortex'	'OPt'
86	'Area 19 of cortex; dorsointermediate part'	'A19DI'
87	'Visual area 2'	'V2'
88	'Visual area 2'	'V2'
89	'Visual area 2'	'V2'
90	'Visual area 3 (ventrolateral posterior area)'	'V3'
91	'Visual area 3 (ventrolateral posterior area)'	'V3'
92	'Temporal area TE; occipital part'	'TEO'
93	'Temporal area TE3'	'TE3'
94	'Visual area 4; transitional part'	'V4T'
95	'Visual area 5 (middle temporal area)'	'V5'
96	'Visual area 4; transitional part'	'V4T'

Monkey O

ch#	Name of cortical areas	Abbrebiations
1	'Temporal area TE3'	'TE3'
2	'Temporal area TE3'	'TE3'
3	'Temporal area TE3'	'TE3'
4	'Temporal area TE3'	'TE3'
5	'Superior temporal rostral area'	'STR'
6	'Superior temporal rostral area'	'STR'
7	'Proisocortical motor region (precentral opercular cortex)'	'ProM'
8	'Auditory cortex; rostrotemporal lateral area'	'AuRTL'
9	'Auditory cortex; rostral parabelt'	'AuRPB'
10	'Temporo-parieto-occipital association area (superior temporal polysensory cortex)'	'TPO'
11	'Fundus of superior temporal sulcus area of cortex'	'FST'
12	'Fundus of superior temporal sulcus area of cortex'	'FST'
13	'Visual area 4 (ventrolatereral anterior area)'	'V4'
14	'Parietal area PE; caudal part'	'PEC'
15	'Medial intraparietal area of cortex'	'MIP'
16	'Lateral intrapartietal area of cortex'	'LIP'
17	'Parietal area PG'	'PG'
18	'Medial superior temporal area of cortex'	'MST'
19	'Medial superior temporal area of cortex'	'MST'
20	'Fundus of superior temporal sulcus area of cortex'	'FST'
21	'Temporo-parieto-occipital association area (superior temporal polysensory cortex)'	'TPO'
22	'Auditory cortex; middle lateral area'	'AuML'
23	'Auditory cortex; primary area'	'AuA1'
24	'Temporoparietal transitional area'	'TPt'
25	'Parietal area PFG'	'PFG'
26	'Parietal area PE'	'PE'
27	'Parietal area PE'	'PE'
28	'Area 3b of cortex (primary somatosensory)'	'A3b'
29	'Areas 1 and 2 of cortex'	'A1/2'
30	'Parietal area PF'	'PF'
31	'Secondary somatosensory cortex; external part'	'S2E'
32	'Auditory cortex; primary area'	'AuA1'
33	'Auditory cortex; middle lateral area'	'AuML'
34	'Temporo-parieto-occipital association area (superior temporal polysensory cortex)'	'TPO'
35	'Auditory cortex; rostral parabelt'	'AuRPB'
36	'Auditory cortex; rostral parabelt'	'AuRPB'
37	'Superior temporal rostral area'	'STR'
38	'Proisocortical motor region (precentral opercular cortex)'	'ProM'
39	'Auditory cortex; rostrotemporal part'	'AuRT'
40	'Auditory cortex; anterolateral area'	'AuAL'
41	'Secondary somatosensory cortex; parietal ventral area'	'S2PV'
42	'Areas 1 and 2 of cortex'	'A1/2'
43	'Area 3b of cortex (primary somatosensory)'	'A3b'
44	'Area 3a of cortex (primary somatosensory)'	'A3a'
45	'Area 4 of cortex; parts a and b (primary motor)'	'A4ab'
46	'Area 6 of cortex; medial (supplementary motor) part'	'A6M'
47	'Area 4 of cortex; parts a and b (primary motor)'	'A4ab'
48	'Area 3a of cortex (primary somatosensory)'	'A3a'
49	'Area 3b of cortex (primary somatosensory)'	'A3b'

50	'Secondary somatosensory cortex; parietal rostral area'	'S2PR'
51	'Areas 1 and 2 of cortex'	'A1/2'
52	'Area 3a of cortex (primary somatosensory)'	'A3a'
53	'Area 8 of cortex; caudal part'	'A8C'
54	'Area 6 of cortex; dorsorostral part'	'A6DR'
55	'Area 6 of cortex; medial (supplementary motor) part'	'A6M'
56	'Area 8b of cortex'	'A8b'
57	'Area 8a of cortex; dorsal part'	'A8aD'
58	'Area 8a of cortex; ventral part'	'A8aV'
59	'Area 6 of cortex; ventral; part a'	'A6Va'
60	'Area 47 (old 12) of cortex; lateral part'	'A47L'
61	'Area 46 of cortex; ventral part'	'A46V'
62	'Area 10 of cortex'	'A10'
63	'Area 10 of cortex'	'A10'
64	'Area 9 of cortex'	'A9'
65	'Visual area 6A (posterior parietal medial area)'	'V6A'
66	'Visual area 6 (dorsomedial area)'	'V6'
67	'Visual area 6 (dorsomedial area)'	'V6'
68	'Visual area 2'	'V2'
69	'Visual area 1'	'V1'
70	'Visual area 1'	'V1'
71	'Visual area 1'	'V1'
72	'Visual area 1'	'V1'
73	'Visual area 1'	'V1'
74	'Visual area 2'	'V2'
75	'Visual area 3 (ventrolateral posterior area)'	'V3'
76	'Visual area 4 (ventrolatereral anterior area)'	'V4'
77	'Visual area 2'	'V2'
78	'Visual area 2'	'V2'
79	'Visual area 1'	'V1'
80	'Visual area 1'	'V1'
81	'Visual area 2'	'V2'
82	'Visual area 2'	'V2'
83	'Visual area 3A (dorsoanterior area)'	'V3A'
84	'Medial intraparietal area of cortex'	'MIP'
85	'Lateral intrapartietal area of cortex'	'LIP'
86	'Visual area 3A (dorsoanterior area)'	'V3A'
87	'Visual area 6 (dorsomedial area)'	'V6'
88	'Visual area 2'	'V2'
89	'Area 19 of cortex; dorsointermediate part'	'A19DI'
90	'Visual area 3 (ventrolateral posterior area)'	'V3'
91	'Visual area 4 (ventrolatereral anterior area)'	'V4'
92	'Temporal area TE3'	'TE3'
93	'Fundus of superior temporal sulcus area of cortex'	'FST'
94	'Visual area 5 (middle temporal area)'	'V5'
95	'Visual area 5 (middle temporal area)'	'V5'
96	'Occipito-parietal transitional area of cortex'	'OPt'

Monkey J

ch#	Name of cortical areas	Abbrebiations
1	'Fundus of superior temporal sulcus area of cortex'	'FST'
2	'Temporo-parieto-occipital association area (superior temporal polysensory cortex)'	'TPO'
3	'Temporal area TE3'	'TE3'
4	'Temporo-parieto-occipital association area (superior temporal polysensory cortex)'	'TPO'
5	'Temporal area TE1'	'TE1'
6	'Superior temporal rostral area'	'STR'
7	'Auditory cortex; rostral parabelt'	'AuRPB'
8	'Auditory cortex; rostral parabelt'	'AuRPB'
9	'Auditory cortex; anterolateral area'	'AuAL'
10	'Medial intraparietal area of cortex'	'MIP'
11	'Lateral intrapartietal area of cortex'	'LIP'
12	'Parietal area PG'	'PG'
13	'Visual area 5 (middle temporal area)'	'V5'
14	'Medial superior temporal area of cortex'	'MST'
15	'Fundus of superior temporal sulcus area of cortex'	'FST'
16	'Temporal area TE3'	'TE3'
17	'Temporal area TE3'	'TE3'
18	'Temporo-parieto-occipital association area (superior temporal polysensory cortex)'	'TPO'
19	'Auditory cortex; middle lateral area'	'AuML'
20	'Auditory cortex; caudolateral area'	'AuCL'
21	'Temporoparietal transitional area'	'TPt'
22	'Parietal area PFG'	'PFG'
23	'Parietal area PE'	'PE'
24	'Areas 1 and 2 of cortex'	'A1/2'
25	'Parietal area PF'	'PF'
26	'Secondary somatosensory cortex; external part'	'S2E'
27	'Auditory cortex; primary area'	'AuA1'
28	'Auditory cortex; primary area'	'AuA1'
29	'Auditory cortex; middle lateral area'	'AuML'
30	'Temporal area TE3'	'TE3'
31	'Temporal area TE3'	'TE3'
32	'Temporo-parieto-occipital association area (superior temporal polysensory cortex)'	'TPO'
33	'Superior temporal rostral area'	'STR'
34	'Superior temporal rostral area'	'STR'
35	'Auditory cortex; rostrotemporal lateral area'	'AuRTL'
36	'Auditory cortex; rostral parabelt'	'AuRPB'
37	'Auditory cortex; rostral area'	'AuR'
38	'Areas 1 and 2 of cortex'	'A1/2'
39	'Area 3b of cortex (primary somatosensory)'	'A3b'
40	'Area 3b of cortex (primary somatosensory)'	'A3b'
41	'Area 3a of cortex (primary somatosensory)'	'A3a'
42	'Area 4 of cortex; parts a and b (primary motor)'	'A4ab'
43	'Area 4 of cortex; parts a and b (primary motor)'	'A4ab'
44	'Area 3a of cortex (primary somatosensory)'	'A3a'
45	'Area 3b of cortex (primary somatosensory)'	'A3b'
46	'Secondary somatosensory cortex; parietal rostral area'	'S2PR'
47	'Proisocortical motor region (precentral opercular cortex)'	'ProM'
48	'Area 6 of cortex; ventral; part b'	'A6Vb'
49	'Area 6 of cortex; ventral; part a'	'A6Va'

50	'Area 6 of cortex; dorsorostral part'	'A6DR'
51	'Area 6 of cortex; dorsorostral part'	'A6DR'
52	'Area 8b of cortex'	'A8b'
53	'Area 8a of cortex; dorsal part'	'A8aD'
54	'Area 8a of cortex; ventral part'	'A8aV'
55	'Area 47 (old 12) of cortex; lateral part'	'A47L'
56	'Proisocortical motor region (precentral opercular cortex)'	'ProM'
57	'Area 47 (old 12) of cortex; medial part'	'A47M'
58	'Area 10 of cortex'	'A10'
59	'Area 47 (old 12) of cortex; lateral part'	'A47L'
60	'Area 47 (old 12) of cortex; lateral part'	'A47L'
61	'Area 46 of cortex; dorsal part'	'A46D'
62	'Area 10 of cortex'	'A10'
63	'Area 9 of cortex'	'A9'
64	'Area 9 of cortex'	'A9'
65	'Visual area 3A (dorsoanterior area)'	'V3A'
66	'Visual area 6 (dorsomedial area)'	'V6'
67	'Visual area 2'	'V2'
68	'Visual area 2'	'V2'
69	'Visual area 1'	'V1'
70	'Visual area 1'	'V1'
71	'Visual area 1'	'V1'
72	'Visual area 1'	'V1'
73	'Visual area 2'	'V2'
74	'Visual area 3 (ventrolateral posterior area)'	'V3'
75	'Visual area 4 (ventrolatereral anterior area)'	'V4'
76	'Visual area 4 (ventrolatereral anterior area)'	'V4'
77	'Visual area 3 (ventrolateral posterior area)'	'V3'
78	'Visual area 2'	'V2'
79	'Visual area 1'	'V1'
80	'Visual area 1'	'V1'
81	'Visual area 2'	'V2'
82	'Visual area 2'	'V2'
83	'Area 19 of cortex; dorsointermediate part'	'A19DI'
84	'Lateral intrapartietal area of cortex'	'LIP'
85	'Occipito-parietal transitional area of cortex'	'OPt'
86	'Visual area 3 (ventrolateral posterior area)'	'V3'
87	'Area 19 of cortex; dorsointermediate part'	'A19DI'
88	'Visual area 2'	'V2'
89	'Visual area 3 (ventrolateral posterior area)'	'V3'
90	'Visual area 4 (ventrolatereral anterior area)'	'V4'
91	'Visual area 4 (ventrolatereral anterior area)'	'V4'
92	'Temporal area TE3'	'TE3'
93	'Temporal area TE3'	'TE3'
94	'Fundus of superior temporal sulcus area of cortex'	'FST'
95	'Visual area 5 (middle temporal area)'	'V5'
96	'Visual area 4; transitional part'	'V4T'

Monkey M

ch#	Name of cortical areas	Abbreviations
1	'Visual area 4 (ventrolatereral anterior area)'	'V4'
2	'Visual area 5 (middle temporal area)'	'V5'
3	'Medial superior temporal area of cortex'	'MST'
4	'Auditory cortex; caudal parabelt area'	'AuCPB'
5	'Auditory cortex; caudal parabelt area'	'AuCPB'
6	'Auditory cortex; rostral area'	'AuR'
7	'Auditory cortex; primary area'	'AuA1'
8	'Auditory cortex; caudomedial area'	'AuCM'
9	'Parietal area PG'	'PG'
10	'Visual area 6A (posterior parietal medial area)'	'V6A'
11	'Medial intraparietal area of cortex'	'MIP'
12	'Lateral intrapartietal area of cortex'	'LIP'
13	'Occipito-parietal transitional area of cortex'	'OPt'
14	'Visual area 4; transitional part'	'V4T'
15	'Visual area 5 (middle temporal area)'	'V5'
19	'Parietal area PG'	'PG'
20	'Parietal area PG'	'PG'
21	'Anterior intraparietal area of cortex'	'AIP'
22	'Parietal area PE'	'PE'
23	'Parietal area PE'	'PE'
24	'Parietal area PE'	'PE'
25	'Parietal area PE'	'PE'
26	'Parietal area PFG'	'PFG'
27	'Parietal area PFG'	'PFG'
28	'Parietal area PG'	'PG'
37	'Auditory cortex; primary area'	'AuA1'
38	'Parietal area PF'	'PF'
39	'Parietal area PF'	'PF'
40	'Areas 1 and 2 of cortex'	'A1/2'
41	'Area 3a of cortex (primary somatosensory)'	'A3a'
42	'Area 4 of cortex; parts a and b (primary motor)'	'A4ab'
43	'Area 3a of cortex (primary somatosensory)'	'A3a'
44	'Area 3b of cortex (primary somatosensory)'	'A3b'
45	'Areas 1 and 2 of cortex'	'A1/2'
46	'Auditory cortex; primary area'	'AuA1'
47	'Auditory cortex; rostral area'	'AuR'
48	'Area 3b of cortex (primary somatosensory)'	'A3b'
49	'Area 3b of cortex (primary somatosensory)'	'A3b'
50	'Area 4 of cortex; parts a and b (primary motor)'	'A4ab'
51	'Area 6 of cortex; dorsocaudal part'	'A6DC'
52	'Area 6 of cortex; dorsorostral part'	'A6DR'
53	'Area 6 of cortex; dorsorostral part'	'A6DR'
54	'Area 6 of cortex; ventral; part a'	'A6Va'
55	'Area 3a of cortex (primary somatosensory)'	'A3a'
56	'Proisocortical motor region (precentral opercular cortex)'	'ProM'
57	'Proisocortical motor region (precentral opercular cortex)'	'ProM'
58	'Area 45 of cortex'	'A45'
59	'Area 6 of cortex; ventral; part b'	'A6Vb'
60	'Area 8a of cortex; ventral part'	'A8aV'

61	'Area 8a of cortex; dorsal part'	'A8aD'
62	'Area 46 of cortex; ventral part'	'A46V'
63	'Area 46 of cortex; dorsal part'	'A46D'
64	'Area 8a of cortex; dorsal part'	'A8aD'
65	'Visual area 6 (dorsomedial area)'	'V6'
66	'Visual area 6 (dorsomedial area)'	'V6'
67	'Visual area 2'	'V2'
68	'Visual area 1'	'V1'
69	'Visual area 1'	'V1'
70	'Visual area 1'	'V1'
71	'Visual area 1'	'V1'
72	'Visual area 1'	'V1'
73	'Visual area 2'	'V2'
74	'Visual area 3 (ventrolateral posterior area)'	'V3'
75	'Visual area 4 (ventrolatereral anterior area)'	'V4'
76	'Visual area 4 (ventrolatereral anterior area)'	'V4'
77	'Visual area 3 (ventrolateral posterior area)'	'V3'
78	'Visual area 2'	'V2'
79	'Visual area 2'	'V2'
80	'Visual area 1'	'V1'
81	'Visual area 2'	'V2'
82	'Visual area 1'	'V1'
83	'Visual area 6 (dorsomedial area)'	'V6'
84	'Visual area 3A (dorsoanterior area)'	'V3A'
85	'Visual area 3A (dorsoanterior area)'	'V3A'
86	'Visual area 6 (dorsomedial area)'	'V6'
87	'Visual area 2'	'V2'
88	'Visual area 2'	'V2'
89	'Visual area 2'	'V2'
90	'Visual area 3 (ventrolateral posterior area)'	'V3'
91	'Visual area 3 (ventrolateral posterior area)'	'V3'
92	'Temporal area TE3'	'TE3'
93	'Fundus of superior temporal sulcus area of cortex'	'FST'
94	'Visual area 5 (middle temporal area)'	'V5'
95	'Visual area 4; transitional part'	'V4T'
96	'Occipito-parietal transitional area of cortex'	'OPt'
