# Supplementary material for: Effects of Ketamine Administration on Auditory Information Processing in the Neocortex of Nonhuman Primates
Source: Front Psychiatry. 2020 Aug 19;11:826. doi: 10.3389/fpsyt.2020.00826 (PMC7466740; doi:10.3389/fpsyt.2020.00826)
Supplement: Supplementary file 3 [file Table_2.pdf]

Monkey J — * FDR adjusted p <0.05; ** p < 0.01

1st Peaks: Amplitude [µV]

PFC	PPC	SM	TC	Au	Vis
-3.8 ± 0.9	-2.8	-4.2 ± 3.1	7.3 ± 1.9	5.1 ± 6.1	-3.1
-3.8 ± 1.4	-2.5	-2.2 ± 2.7*	4.9 ± 2.7	5.5 ± 4.6	NaN
-3.3 ± 0.5	-1.2	-5.1 ± 3.0	12.6 ± 2.9**	6.8 ± 7.0	NaN
-3.6 ± 1.1	NaN	-3.7 ± 3.3	8.3 ± 2.2	4.5 ± 5.3	NaN

Latency [ms]

PFC	PPC	SM	TC	Au	Vis	All
28.1 ± 1.4	24	36.8 ± 4.1	37.8 ± 1.4	31.3 ± 8.4	39	33.7 ± 6.2
32.9 ± 1.2**	27	43.4 ± 8.9	44.0 ± 4.5**	37.0 ± 11.2	NaN	39.4 ± 9.0**
32.6 ± 2.1**	19	40.5 ± 5.9	44.9 ± 2.8**	35.2 ± 8.7	NaN	38.0 ± 7.6*
27.9 ± 2.8	NaN	38.7 ± 6.8	39.9 ± 1.6*	31.9 ± 9.2	NaN	34.5 ± 7.6

2nd Peaks: Amplitude [µV]

PFC	PPC	SM	TC	Au	Vis
0.35 ± 0.9	-2.01 ±4.2	1.35 ± 2.1	-2.95 ± 1.4	-2.10 ± 2.5	2.98
4.64 ± 0.6**	0.41 ± 5.6	0.02 ± 4.7	-7.40 ± 1.7**	-9.53 ± 4.6**	2.62
9.72 ± 2.3**	-0.52 ± 7.4	2.46 ± 5.4**	-11.21 ± 5.8**	-8.70 ± 8.8*	6.21
6.97 ± 1.9**	-1.13 ± 5.1	3.25 ± 5.1**	-5.92 ± 0.8**	-4.84 ± 4.2*	4.59

Latency [ms]

PFC	PPC	SM	TC	Au	Vis	All
54.7 ± 5.7	47.7 ± 15.9	74.3 ± 16.9	83.8 ± 3.3	64.7 ± 17.5	68	68.2 ± 17.0
77.0 ± 1.9**	57.5 ± 27.6	87.6 ± 6.1	91.3 ± 2.1**	77.2 ± 7.1	83	81.0 ± 11.7**
72.0 ± 1.5**	53.0 ± 15.6	86.1 ± 17.2	84.4 ± 3.0	70.6 ± 15.2	76	76.2 ± 14.9
56.3 ± 0.5	49.0 ± 19.1	79.7 ± 22.6	83.5 ± 6.9	63.6 ± 15.8	73	69.1 ± 19.5

Monkey O — * FDR adjusted p <0.05; ** p < 0.01

1st Peaks: Amplitude [µV]

PFC	PPC	SM	TC	Au	Vis
-4.7 ± 0.7	-5.2	-3.2 ± 4.1	9.4 ± 9.9	9.2 ± 5.3	NaN
-3.6 ± 0.3	-6.3	-3.0 ± 5.2	12.9 ± 13.2	15.9 ± 9.2	NaN
-5.4 ± 0.6	-6.6	-4.2 ± 4.9	13.1 ± 9.7	12.4 ± 6.3	NaN
-3.7 ± 0.7	-6.9	-4.2 ± 5.0	10.5 ± 12.9	11.2 ± 6.1	NaN

Latency [ms]

PFC	PPC	SM	TC	Au	Vis	All
22.0 ± 1.3	40	33.1 ± 10.2	34.6 ± 10.0	30.8 ± 6.1	NaN	30.6 ± 8.7
26.3 ± 1.5*	40	34.4 ± 10.7	42.0 ± 1.8	31.9 ± 6.6	NaN	34.0 ± 8.4
27.8 ± 1.3**	45	37.1 ± 10.7	40.2 ± 11.0	35.3 ± 7.9	NaN	35.4 ± 9.2
22.5 ± 2.3	36	32.3 ± 8.4	38.3 ± 1.3	30.4 ± 5.9	NaN	30.6 ± 7.5

2nd Peaks: Amplitude [µV]

PFC	PPC	SM	TC	Au	Vis
5.3 ± 0.6	2.4	0.7 ± 6.2	-7.6 ± 5.7	-10.7 ± 6.6	NaN
13.6 ± 0.7**	NaN	-0.1 ± 12.7	-1.6 ± 10.3	-26.1 ± 19.2	NaN
11.8 ± 0.2**	5.3	1.6 ± 10.7	-9.2 ± 15.6	-22.4 ± 16.3	NaN
5.4 ± 1.4	4.4	2.7 ± 10.8*	-6.0 ± 9.7	-15.1 ± 8.7	NaN

Latency [ms]

PFC	PPC	SM	TC	Au	Vis	All
50.2 ± 2.8	71	72.6 ± 19.7	92.6 ± 32.4	77.1 ± 23.7	NaN	72.6 ± 24.3
72.8 ± 1.5**	NaN	70.1 ± 5.2	72.8 ± 18.5	71.4 ± 7.6	NaN	71.6 ± 8.3
80.0 ± 2.3**	97	72.0 ± 4.7	86.0 ± 29.6	79.3 ± 13.2	NaN	78.9 ± 12.8
56.8 ± 13.8	70	61.5 ± 5.5	67.3 ± 24.4	71.3 ± 12.6	NaN	64.7 ± 13.6

Monkey M — * FDR adjusted p <0.05; ** p < 0.01

1st Peaks: Amplitude [µV]

PFC	PPC	SM	TC	Au	Vis
NaN	-1.6 ± 1.9	2.3 ± 3.9	NaN	11.7 ± 9.1	-2.8 ± 1.1
NaN	-1.3 ± 1.4	3.5 ± 3.7	NaN	10.8 ± 5.6	-1.8 ± 0.4
NaN	-1.7 ± 1.7	1.2 ± 3.7	NaN	12.0 ± 8.5	-2.1 ± 0.4
NaN	-1.7 ± 1.7	2.7 ± 3.1	NaN	15.3 ± 6.2	-2.6 ± 0.6

Latency [ms]

PFC	PPC	SM	TC	Au	Vis	All
NaN	27.7 ± 5.6	26.7 ± 8.2	NaN	31.4 ± 6.2	24.0 ± 3.0	27.4 ± 6.0
NaN	36.1 ± 2.3**	33.0 ± 3.4	NaN	37.3 ± 7.4	36.2 ± 1.1**	35.7 ± 3.5**
NaN	30.7 ± 3.7	34.0 ± 8.8	NaN	33.4 ± 4.5	28.8 ± 2.8	31.5 ± 5.1*
NaN	24.4 ± 2.1	21.0 ± 1.0	NaN	27.0 ± 4.7	22.3 ± 0.5	24.0 ± 3.0

2nd Peaks: Amplitude [µV]

PFC	PPC	SM	TC	Au	Vis
NaN	1.6 ± 2.8	-4.9 ± 8.1	NaN	-7.5 ± 9.6	3.3 ± 1.7
NaN	1.7 ± 2.8	-10.9 ± 10.5	NaN	-19.4 ± 14.8	4.0 ± 1.0
NaN	2.9 ± 3.9	-9.7 ± 11.1	NaN	-13.8 ± 15.9	5.9 ± 1.7
NaN	3.0 ± 3.8	-6.0 ± 10.0	NaN	-14.5 ± 13.5	5.0 ± 1.6

Latency [ms]

PFC	PPC	SM	TC	Au	Vis	All
NaN	58.8 ± 5.0	57.7 ± 2.9	NaN	63.8 ± 3.8	57.3 ± 2.6	59.1 ± 4.5
NaN	79.9 ± 5.4**	79.2 ± 9.7**	NaN	82.8 ± 2.6**	83.8 ± 5.1**	81.0 ± 6.0**
NaN	65.6 ± 2.7**	64.3 ± 7.7	NaN	68.6 ± 5.9	64.5 ± 1.6**	65.6 ± 4.6**
NaN	55.6 ± 3.8	51.1 ± 2.3**	NaN	60.4 ± 6.3	54.7 ± 3.1	55.2 ± 4.7**

Supplementary Table S2. The amplitudes and latencies of first and second peaks of AERs of LF in six electrodes groups based on putative cortical areas.
